# Sustained Release from Injectable Composite Gels Loaded with Silver Nanowires Designed to Combat Bacterial Resistance in Bone Regeneration Applications

**DOI:** 10.3390/pharmaceutics11030116

**Published:** 2019-03-12

**Authors:** Arianna De Mori, Meena Hafidh, Natalia Mele, Rahmi Yusuf, Guido Cerri, Elisabetta Gavini, Gianluca Tozzi, Eugen Barbu, Mariateresa Conconi, Roger R. Draheim, Marta Roldo

**Affiliations:** 1 School of Pharmacy and Biomedical Science, University of Portsmouth, St Michael’s Building, White Swan Road, Portsmouth PO1 2DT, UK; arianna.demori@port.ac.uk (A.D.M.); meena.hafidh@myport.ac.uk (M.H.); natalia.mele.nm@gmail.com (N.M.); rahmi.yusuf@port.ac.uk (R.Y.); eugen.barbu@port.ac.uk (E.B.); roger.draheim@port.ac.uk (R.R.D.); 2 Department of Architecture, Design and Urban Planning—GeoMaterials Lab, University of Sassari, Via Piandanna 4, 07100 Sassari, Italy; gcerri@uniss.it; 3 Department of Chemistry and Pharmacy, University of Sassari, via Muroni 23, 07100 Sassari, Italy; eligav@uniss.it; 4 Zeiss Global Centre, School of Engineering, University of Portsmouth, Anglesea Building, Anglesea Road, Portsmouth PO1 3DJ, UK; gianluca.tozzi@port.ac.uk; 5 Department of Pharmaceutical and Pharmacological Sciences, University of Padova, via Marzolo 5, 35131 Padova, Italy; mariateresa.conconi@unipd.it

**Keywords:** chitosan hydrogels, silver nanowires, controlled release, antimicrobial activity, bone regeneration

## Abstract

One-dimensional nanostructures, such as silver nanowires (AgNWs), have attracted considerable attention owing to their outstanding electrical, thermal and antimicrobial properties. However, their application in the prevention of infections linked to bone tissue regeneration intervention has not yet been explored. Here we report on the development of an innovative scaffold prepared from chitosan, composite hydroxyapatite and AgNWs (CS-HACS-AgNWs) having both bioactive and antibacterial properties. In vitro results highlighted the antibacterial potential of AgNWs against both gram-positive and gram-negative bacteria. The CS-HACS-AgNWs composite scaffold demonstrated suitable Ca/P deposition, improved gel strength, reduced gelation time, and sustained Ag^+^ release within therapeutic concentrations. Antibacterial studies showed that the composite formulation was capable of inhibiting bacterial growth in suspension, and able to completely prevent biofilm formation on the scaffold in the presence of resistant strains. The hydrogels were also shown to be biocompatible, allowing cell proliferation. In summary, the developed CS-HACS-AgNWs composite hydrogels demonstrated significant potential as a scaffold material to be employed in bone regenerative medicine, as they present enhanced mechanical strength combined with the ability to allow calcium salts deposition, while efficiently decreasing the risk of infections. The results presented justify further investigations into the potential clinical applications of these materials.

## 1. Introduction

Over the last few decades, significant advancements in the synthesis and characterization of one dimensional (1D) nanostructured materials have led to novel applications in various fields such as material science, energy technologies, engineering and biomedical science. A wide range of nanostructures, such as nano-tubes, nano-fibres, nano-filaments, nano-whiskers, nano-horns, nano-needles, nano-ribbons and nano-wires are classified as 1D nanostructures [1] that are all characterized by a high aspect ratio [2]. These structures present remarkable variation in their electrical, optical and magnetic properties when compared to the bulk materials due to their reduced dimensions, controlled structure and large surface-to-volume ratio [3].

One, two, and three-dimensional silver nanostructures have been previously reported, with many studies focusing on silver nanowires (AgNWs). AgNWs are anisotropically grown to obtain an aspect ratio higher than 10 and are typically 10 nm–200 nm in diameter and 5 µm–100 µm in length [2]. To date, silver nanoparticles (AgNPs) have been much more extensively studied than AgNWs, and their distinctive high thermal and electrical conductivity, surface-enhanced Raman scattering, chemical stability, catalytic activity and non-linear optical behavior have been highlighted; as well as their antibacterial activity that has extended their usage into several biomedical fields [4]. They are promising agents against a wide range of fungi, viruses and bacterial species. Even though the mechanism of action is still not entirely understood, it is believed that nanosilver serves as a source for Ag^+^ ions that can rupture microbial cell walls, denature proteins, block cell respiration and eventually induce cell death [5]. AgNPs have been used as antimicrobial agents in wound dressing [6], the coating of catheters [7] and cardiovascular implants [8], bone cements [9] and dental materials [10]. Currently, literature on the biomedical applications of AgNWs is still limited [2]. Emerging applications include textiles [11], wound coatings [12], drug release and tissue regeneration [13].

While the antimicrobial activity of AgNWs in poly(vinylpyrrolidone) fibres has been just recently reported [14], a few more biomedical applications of AgNWs are also being considered [2]. In this paper we explore for the first time the potential use of AgNWs in composite materials designed to prevent infection in bone regeneration applications, as they can provide advantages such as biocompatibility, bioactivity and a 3D structure that can be employed as a supporting scaffold. Bone defects remain a major problem in orthopedics and due to the deficiencies of current commercially available bone grafts, alternative materials are needed [15]. Hydrogels are very good candidates to overcome these obstacles since they are 3D, hydrophilic, crosslinked polymeric networks that resemble the extracellular matrix with a porosity and aqueous environment that allow the transportation of substances such as nutrients. The main challenge within the field is to produce a bioactive and non-toxic hydrogel scaffold with sufficient mechanical strength [16,17]. Chitosan (CS), a polysaccharide that is biocompatible, biodegradable and possesses antibacterial properties has been employed to formulate such scaffolds, however, it has poor mechanical properties and inferior bone induction capability [17]. Our previous work has focused on the development of reinforced bioactive CS scaffolds containing hydroxyapatite (HA), which favors biomineralization of the scaffold, and carbon nanotubes that provide mechanical enhancement [18]. The present work aims at substituting carbon nanotubes with other 1D nanostructures, namely AgNWs, that afford the two-fold advantage of mechanically reinforcing the hydrogel structure while enhancing its antibacterial properties. In fact, the success of bone-defect surgical treatment can often be compromised by bacterial infection that may result in chronic inflammation, implant failure and even death [19,20]. To date, few studies have been carried out to evaluate the antibacterial properties of AgNWs alone [2], and very limited work has been done in the investigation of their activity within composite materials. Here, we present the synthesis via the polyol method and the full characterization of AgNWs, followed by the in vitro characterization of the composite hydrogels containing the nanowires with particular emphasis on the antimicrobial, bioactive and biocompatible properties of the scaffolds.

## 2. Materials and Methods

Di-potassium hydrogen phosphate anhydrous and OCT cryostat embedding medium (Tissue Tek^®^) were obtained from BDH (VWR, Lutterworth, UK). Glutamax and BCA Protein Assay Reagent Kit were obtained from Thermo Fisher (Basingstoke, UK). An Alkaline Phosphatase Assay kit was purchased from Abcam (Cambridge, UK). An ATPlite Luminescence ATP Detection Assay System was purchased from PerkinElmer (Coventry, UK). The osteoblastic cell line MC3T3-E1 was purchased from DSMZ (Leibniz-Institut DSMZ-Deutsche Sammlung von Mikroorganismen und Zellkulturen GmbH, Braunschweig, Germany). The Alpha-minimal essential medium (α-MEM) was purchased from Life Technologies (Baltimore, MD, USA). Poly(vinylpyrrolidone) powder (PVP, 55 kDa), chitosan from shrimp shells low viscosity (M_n_ 149.9 ± 4.7 kDa, M_w_ 170.5 ± 4.9 kDa as determined by GPC-MALLS; degree of deacetylation ~85%, calculated by ^1^H-NMR) were sourced from Sigma-Aldrich (Irvine, UK). All the other materials, unless specified above, were from Sigma-Aldrich (Irvine, UK) and Fisher (Loughborough, UK).

### 2.1. Synthesis and Physicochemical Characterisation of Silver Nanowires (AgNWs)

AgNWs were synthesized via the polyol method, using silver nitrate (AgNO_3_) as the Ag source [21]. PVP (3 g) was fully dissolved in glycerol (95 mL) by heating to 80 °C. The solution was cooled down and AgNO_3_ (0.79 g) was added under vigorous stirring (800 rpm) until the powder was fully dissolved. NaCl (5 mM in final solution) was mixed into 10 mL of glycerol and added to the PVP/glycerol solution. The reaction temperature of the mixture was gradually raised to 210 °C. Once the temperature was reached, it was maintained for a further 10 min. Samples (1 mL) were taken at different temperatures, diluted (1:1) with water and analyzed by UV (300–900 nm, Thermo Scientific Nicolet Evolution 100 UV-Visible Spectrophotometer, Thermo Fisher Scientific, Rochford, UK) to follow the formation of the AgNWs. Finally, the reaction was cooled down to room temperature, diluted 1:1 with deionized water and centrifuged (4000 rpm, 1 h), the pellet was washed twice with isopropanol (4000 rpm, 30 min) and twice with deionized water (4000 rpm, 10 min). The product was stored in deoxygenated purified water at room temperature, protected from light or freeze dried. Freeze dried AgNWs were suspended in a chitosan solution (1%, in lactic acid 0.1 M) to achieve different concentrations (0.5–1 mg/mL), by vortexing and sonicating for several minutes. These were spread onto SEM specimen stubs and dried for two days under vacuum. Samples were gold-coated with a Polaron e500 (Quoram Technologies, Lewes, UK) sputter coater and analyzed with a Jeol JSM-6160L scanning electron microscope (Jeol, Welwyn Garden City, UK) with electron backscatter. Images were analyzed with ImageJ (NIH, Madison, WI, USA) to determine the size distribution of the wires obtained. Each individual AgNW identified in the pictures was measured 3 times. Freeze-dried products were suspended in a chitosan solution (1%, in lactic acid 0.1 M), to achieve different concentrations (0.5–1.5 mg/mL), by vortexing and sonicating for several min. Samples were then dropped onto TEM grids (Agar scientific square mesh TEM support grids-copper) and allowed to dry at room temperature and normal pressure. Dried grids were stored in Petri dishes in a desiccator until observation using a Jeol JEM 2100 Transmission Electron Microscope (Jeol, Welwyn Garden City, UK). The crystal structure of AgNWs was determined with a Bruker D2-Phaser diffractometer (Bruker, Milan, IT). Instrumental parameters were: CuKα radiation, 30 kV, 10 mA, LynxEye PSD detector with an angular opening of 5°, 2θ range 6–84°, step size 0.020°, time per step 2 s, spinner 15 rpm. The alignment of the instrument was calibrated using an international standard (NIST 1976b). A low-background silicon crystal specimen holder (Bruker) was used. The analysis was performed at room temperature (25 °C). The XRD pattern was evaluated using the software Bruker EVA 14.2 (DIFFRACplus Package, Bruker, Milan, IT) coupled with the database PDF-2 (ICDD). Surface zeta potential was measured using a Malvern Zetasizer instrument (Nano ZS, UK) using nanoparticles suspended by sonication in deionized water. An average of twelve measurements per sample were carried out.

### 2.2. Formulation and Characterization of Hydrogels

Chitosan based hydrogels were formulated according to the composition listed in Table 1, following the method previously described by Cancian et al. [18]. The full physicochemical characterization of HACS has been previously reported [17]. The freeze dried scaffolds were stored in a desiccator until further use. Gelation time was assessed using the inversion test, as described by Ganji et al. [22]. Formulations were prepared in 2 mL aliquots; after the addition of GP, the composites were vortexed (2 min) and stored at 4 °C for 12 h to remove air bubbles. The vials were then incubated at 37 °C in a temperature controlled bath (Grant, SUB Aqua Pro, Shepreth, UK). The sol-gel transition was determined by inverting the vials; the time at which the gel stopped flowing was recorded as the gelation time.

Texture profile analysis was performed using a texture analyzer (XT plus, Stable Micro Systems Ltd., Godalming, UK) by driving a Delrin (polyformaldehide) probe (10 mm diameter) into the gels at 1 mm/s and to a depth of 5 mm; six measurements were taken at room temperature before and after the sol/gel transition took place. Force/distance curves were obtained and the maximum force value recorded was reported as gel strength (N).

An equilibrium of swelling was determined on freeze-dried hydrogels that were weighed and allowed to swell in phosphate buffer solution (PBS, 15 mL, pH = 7.4) at 37 °C. At different time points, the hydrogels were removed from the buffer and weighed, after removing excess liquid. The experiment (carried out in triplicate) was continued until equilibrium was reached. The swelling ratio (*Q*) was calculated according to Equation (1):

(1) $$Q\left(\%\right)=\frac{\left(W_{s}-W_{d}\right)\times 100}{W_{d}}$$

where *W_s_* and *W_d_* represent the weight of the swollen and dry state samples, respectively.

The porosity of the scaffolds was determined on dried gels according to the method described by Nanda et al. [23]. The in vitro degradation of pre-weighed dried scaffolds was carried out in 5 mL sterile phosphate-buffered solution (PBS, pH 7.4) containing 1.0 mg/mL lysozyme. The PBS/lysozyme solution was changed every 3–4 days. Samples were incubated at 37 °C with gentle mechanical agitation for the period of study (80 rpm). After 7, 14 and 21 days, samples were removed from the medium, rinsed with distilled water, freeze dried and weighed. The experiment was carried out in triplicate. The extent of in vitro degradation was calculated using Equation (2):

(2) $$Mass\;loss\left(\%\right)=\left[\left(W_{i}-W_{d}\right)/W_{i}\right]\times 100$$

where *W_i_* is the initial weight of the scaffold and *W_d_* is the weight of the scaffold after the degradation experiment.

In vitro calcification studies were performed to investigate the ability of the composite hydrogels to induce calcium salts deposition. The freeze dried samples were submerged into 15 mL of simulated body fluid (SBF, prepared according to Kokubo and Takadama [24]) and incubated at 37 °C for 7, 14 and 21 days; the SBF was changed every four days. Finally, samples were freeze dried. Controls were hydrated in deionized water for 5 h and dried as above. All samples were analyzed by SEM (as described above), coupled with EDS (Silicon Drift Detector (SDD)—X-MaxN, Oxford Instruments, UK). EDS images for calcium (Ca) and phosphorus (P) were analyzed using ImageJ 3D Viewer after being overlaid to corresponding SEM images. The total area of overlapping for Ca and P onto the total sample surface area was used to calculate percentage of Ca and P salt deposition (n = 3). In order to further explore the overall salt deposition within the scaffolds micro-CT was used. Non coated scaffolds were scanned with the microCT scanner (Versa 510, Zeiss, Dublin, CA, USA) set to a voltage of 40 kV and a current of 76 μA. An isotropic voxel size of 4.077 μm and exposure of 3 s were used. The images were then analyzed using ImageJ (v1.50, NIH, Madison, WI, USA) as described by Cancian et al. [18].

### 2.3. Ag^+^ Release Studies

Dried AgNWs and freeze-dried hydrogels were soaked in HPLC-grade water and incubated at 37 °C, with shaking (90 rpm). At scheduled time points, the AgNWs in water were centrifuged at 2000 rpm for 3 min (this step was not required for CS-HACS-AgNWs) and 1 mL of supernatant was taken and replaced with 1 mL of fresh water. The concentration of silver ions was determined by a furnace atomic absorption spectro-photometer (Varian SpectrAA 220FS, Varian Medical Systems, Crawley, UK) at a wavelength and spectral bandwidth of 328.1 nm and 0.2 nm, respectively. The experiment was carried out in triplicate.

### 2.4. Antimicrobial Activity of AgNWs

The antibacterial activity of AgNWs was examined by a suspension assay against gram-negative *Escherichia coli* (ATCC 25922) and gram-positive *Staphylococcus aureus* (ATCC 25923), Methicillin-resistant *Staphylococcus aureus* (ATCC 12403) and *Staphylococcus saprophyticus* (ATCC 15305). The bacteria were transferred from −80 °C (15% glycerol) into 5 mL of fresh sterile LB medium by a sterile toothpick and incubated (at 37 °C and 200 rpm) until the bacterial suspension was cloudy (one day for *E. coli* and *S. aureus*, two days for MRSA and three days for *S. saprophyticus*). Then, 50 µl of bacterial suspension was transferred into 5 mL of fresh sterile LB medium and the bacteria were further incubated at 37 °C until the suspension was newly cloudy (one day for *E. coli*, *S. aureus* and MRSA and two days for *S. saprophyticus*). Cells at a concentration of 1 × 10^6^ CFU/mL (equal to an OD_600nm_ of 0.001, MaxQ™ 8000, Thermo Scientific) were grown in 50 mL of liquid LB medium supplemented with 12.5, 25, 50 and 100 µg/mL of AgNWs. The stock suspensions of AgNWs were prepared the day before use and stored at 4 °C. This was accomplished by firstly weighing the dried AgNWs and finally resuspending them in sterile LB by sonication (40 kHz, CamLab, Cambridge, UK) until the suspension was homogeneous. This step ensured the sterilization of the AgNWs suspension due to high frequency ultrasounds. Pure medium with bacterial cell inoculation and pure LB served as controls. Growth rates and bacterial concentrations were detected by measuring the optical density of the inoculated LB broth medium at 600 nm at different time points. Different concentrations (12.5, 25, 50 and 100 µg/mL) of the stock AgNWs suspension and pure LB were used as blanks. The experiment was carried out into autoclaved 250 mL glass flasks at 37 °C and 200 rpm, in triplicate.

### 2.5. Antimicrobial Activity of Composite Hydrogels

The antibacterial activity of CS-HACS and CS-HACS-AgNWs scaffolds was examined by a suspension assay against the micro-organisms listed in Section 2.4. The scaffolds were sterilized by firstly submerging them in 70% (*v*/*v*) ethanol for 15 min and then rinsing them three times with sterile PBS. Finally, they were autoclaved in LB and sonicated for 15 min at 40 kHz before discarding the LB. All the scaffolds were then placed into 2 mL of 1 × 10^5^ CFU/mL cell suspension in glass vials and analyzed as described below.

#### 2.5.1. Biofilm Formation Assay

For the determination of biofilm formation, after incubation at 37 °C and 100 rpm for 24 h, the hydrogels were gently soaked in PBS for 10 s to remove loosely adherent bacteria and subsequently fixed in paraformaldehyde (4% in PBS) overnight, before being washed in PBS (3 times) and soaked into 5%, 10%, 20% (2 h) and 30% (overnight) sucrose solutions. The scaffolds were transferred into aluminum cases and embedded into optimal cutting temperature compound (OCT). Then, they were rapidly frozen in pentane, submerged into liquid nitrogen. Hydrogels were stored at −80 °C until cryosectioning. Hydrogels were cut into 7–9 μm slices that were placed onto poly-l-lysine coated slides. The sections were air-dried for 30 min and stored at −20 °C until staining. Prior to staining, the sections were washed with deionized water for 1 h and stained with carbol fuchsin (for gram-negative cells) or crystal violet (for gram-positive cells). Slides were stained for 1 min and then washed with running deionized water for 5 min. The slides were left to dry and observed with an optical microscope (GXM-L1500BHTG, GTVision, Sudbury UK).

#### 2.5.2. Antimicrobial Testing of the Dried Hydrogels

To test the antimicrobial properties of the scaffolds, these were prepared as above but samples of the incubation medium were taken at scheduled times (3, 7 and 24 h), the bacterial suspensions were transferred into a sterile 15 mL tube and the scaffolds were washed with sterile PBS (1 mL) vortexing for 30 s. The last step was done for collecting the not fully attached bacteria. The PBS solution was then transferred into the 15 mL tube. The total collected bacterial solution was serially diluted in PBS (from 10^−1^ to 10^−15^) into sterile 2 mL test tubes and plated in triplicate on LB agar plates (100 µL per plate). After static incubation for 18 h at 37 °C (HeraTherm Incubator, Thermo Scientific) the colony forming units (CFUs) were manually counted. Controls were prepared with: LB only, scaffolds and LB, and individual bacteria in LB only.

### 2.6. Cell Culture Studies

The osteoblastic cell line MC3T3-E1 was cultured with proliferation medium composed of α-MEM containing 10% fetal bovine serum and 1% penicillin/streptomycin. Cells were grown at 37 °C in a humid atmosphere with 5% CO_2_. The medium was refreshed three times a week. Freeze-dried hydrogels were hydrated for 5 min, then immersed in 70% ethanol, washed with phosphate buffer saline (PBS) four times, transferred into a 24-well plate, and irradiated with UVA light overnight. Samples were equilibrated in proliferation medium for 1 h, the medium was then removed and 1.8 × 10^4^ cells were seeded in each well in osteogenic differentiation medium which contained α-MEM without nucleosides, 10% FBS, 1% penicillin/streptomycin, 1% glutamax, 10 nM β-glycerophosphate and 50 µg/mL ascorbic acid. Cultures grown on tissue-culture polystyrene plates were used as control. After 7 and 14 days from seeding, cell proliferation, and differentiation were evaluated. Cell viability was assessed using the Luminescence ATP Detection Assay according to the manufacturer’s instructions. Briefly, substrate solution was added to cell lysates and the luminescence was measured with VICTOR3 1420 Multilabel Counter (PerkinElmer, Coventry, UK). Counts per seconds were converted in cell number by using a standard curve previously obtained with known cell numbers (from 10^4^ to 10^5^ cells). The detection of ALP activity was carried out by using the Alkaline Phosphatase Assay kit, following the manufacturer’s instructions. Briefly, cell lysates were centrifuged and supernatants were treated with 5 mM *p*-nitrophenyl phosphate in the dark. Optical density (OD) was read at 405 nm by using the EL 311 SX microplate autoreader (BioTek Instruments, Inc., Winooski, VT, USA). OD values were converted in nanomoles of ALP by using a standard curve previously obtained with known ALP quantities (from 4 to 20 nanomoles). The protein content of each sample was analyzed with a BCA kit. Finally, results were expressed as nM ALP/µg protein.

### 2.7. Statistical Analysis

Statistical analysis has been performed for all experiments and is detailed in each figure caption.

## 3. Results

### 3.1. Synthesis and Physicochemical Characterization of Silver Nanowires

**Figure 1:**
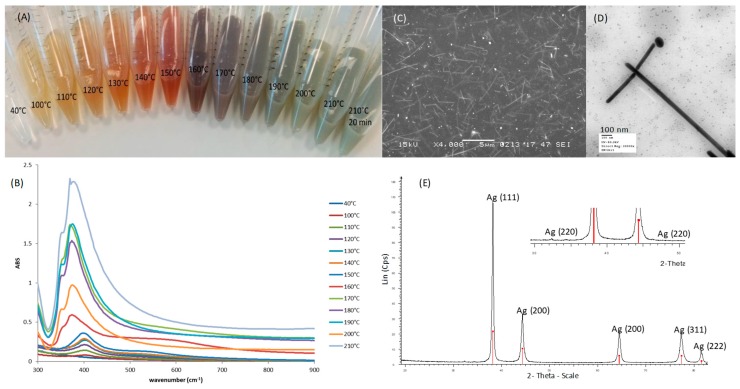
Characterization of silver nanowires-AgNWs: (**A**) Typical color change of the reaction mixture at different temperatures; (**B**) the corresponding Ultra Violet spectra; (**C**) Scanning Electron Microscopy image of AgNWs suspended in a solution of chitosan in lactic acid (scale bar 4 µm); (**D**) Transmission Electron Microscopy image of AgNWs (scale bar 100 nm); (**E**) X Ray Diffraction pattern of the synthesized AgNWs (red bars: Ag, PDF No. 04-0783; blue bars: AgCl, PDF No. 31-1238).

AgNWs were successfully synthesized using the polyol method. During the synthesis, as the temperature was increased, a typical change in color from clear to yellow, then orange, red, grey and green was observed (Figure 1A). This correlated to the changes in the UV-Vis spectrum with increasing temperatures (Figure 1B). Below 180 °C a peak was present at 410 nm, this is due to the surface plasmon resonance (SPR) signal of Ag nanoparticles and nanorods that initially form as nucleation sites for the growth of nanowires. As the temperature increases, peaks appear at 350 nm and 380 nm, these are attributed to the formation of Ag nanowires, as confirmed by SEM and TEM (Figure 1C,D) [25]. The XRD pattern (Figure 1E) confirmed the formation of face-centered cubic metallic Ag. In fact, the d-values perfectly matched to PDF Number 04-0783 (Silver-3C, syn); also the intensity of the peaks corresponded, except for (111). In the card 04-0783, the (111)/(200) intensity ratio is 2.5, whereas in our pattern it was 3.4 indicating the formation of well-elongated AgNWs [26,27]. A minimal trace impurity of AgCl (PDF No. 31-1238, Chlorargyrite) can be seen at 2θ 32.2° and 46.2° (Figure 1E), an aspect sometimes reported in the synthesis of AgNWs [28]. Thus, our AgNWs synthesis product was mainly composed of metallic silver, even though small traces of ionic silver were found, indicating that both the synthesis and the washing steps were efficient enough to guarantee a nearly pure product. We also studied the morphology of AgNWs obtained at the end of the synthesis. They had an average length of 5.03 ± 1.85 µm and an average diameter of 99.45 ± 20.20 nm (See Supplementary Figure S1 online), as determined by analyzing SEM images with ImageJ. These dimensions are in good agreement with those reported by Yang et al. [21]. The zeta potential of AgNWs in deionized water was found to be −10.88 ± 1.86 mV, indicating their tendency to aggregate in aqueous media.

### 3.2. Characterization of Composite Gels

**Figure 2:**
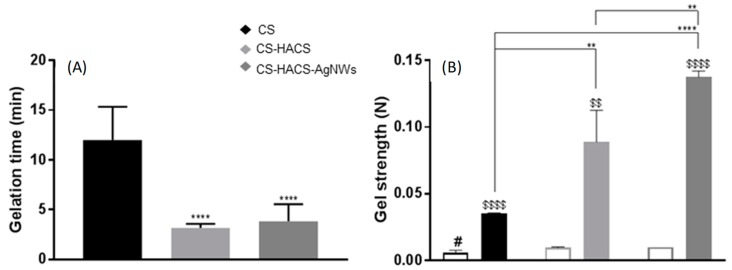
Physical characterization of the hydrogels: Chitosan (CS, black), chitosan gel with chitosan hydroxyapatite composite (CS-HACS, light grey) and chitosan gel with chitosan hydroxyapatite composite and silver nanowires (CS-HACS-AgNWs, dark grey). (**A**) Gelation time calculated by the inverted tube method. Data are reported as mean ± SD (n = 9). One-way Analysis of Variance (ANOVA) returned *p* < 0.0001. Post-hoc Tukey’s comparison test results are shown in the graph: **** indicates *p* < 0.0001 as compared to the control (CS); (**B**) Gel strength calculated by texture analysis before (empty bars) and after (filled bars) gelation. Data are represented as mean ± SD (n = 3). The *t*-test performed on all samples before and after gelation showed significantly different strength values for all gels (^$$^ indicates *p* < 0.01 and ^$$$$^ *p* < 0.0001). One-way ANOVA to compare the different formulations returned *p* < 0.01 before and *p* < 0.0001 after gelation. Post-hoc Tukey’s test results showed that before gelation only CS is different from all other gels (^#^ *p* < 0.05), while the individual results for after gelation are reported on the graph (** *p* < 0.01; **** *p* < 0.0001).

AgNWs were used to formulate composite chitosan gels; the thermosensitive gels were composed of chitosan crosslinked with glycerol phosphate and mixed with a chitosan/hydroxyapatite composite (HACS). As previously described, full physicochemical characterization can be found in [17,18] and SEM images are available in the supplementary information (Figure S2). A significant reduction in the time of sol/gel transition was observed on the addition of the HACS composite to the chitosan gel (from 12.0 ± 3.3 min to 3.2 ± 0.4 min; *p* < 0.0001; Figure 2A), no further significant change was observed on the addition of AgNWs. When only AgNWs were added, a non-significant (*p* > 0.05) reduction to 10.3 ± 2.4 min was observed (see Supplementary Figure S3 online). On the other hand, AgNWs induced a further significant increase in gel strength (to 0.138 ± 0.005 N, *p* < 0.01) after the increase initially observed on addition of HACS (from 0.035 ± 0.001 N to 0.089 ± 0.024 N, *p* < 0.01; Figure 2B). Also, AgNWs alone were able to significantly increase the strength of the chitosan gel (to 0.136 ± 0.001 N, *p* < 0.0001, see Supplementary Figure S4 online).

**Figure 3:**
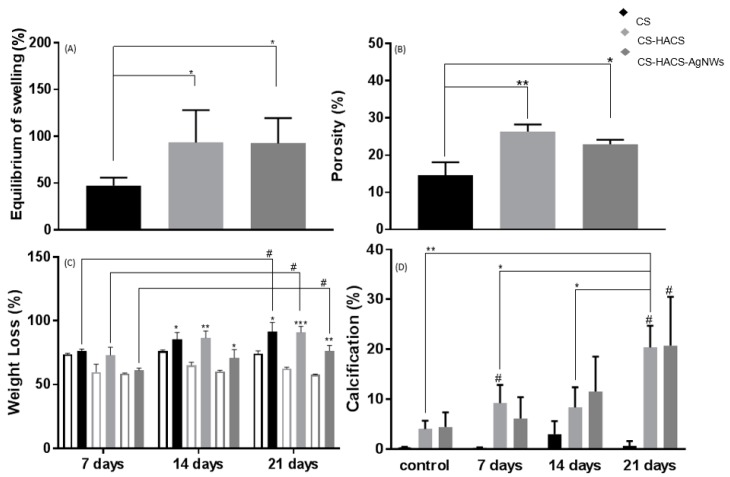
Further physical characterization, enzymatic degradation and bioactivity determination of hydrogels: CS (black), CS-HACS (light grey) and CS-HACS-AgNWs (dark grey). (**A**) Equilibrium swelling (Q%), data are reported as mean ± SD (n = 6). One-way ANOVA returned *p* < 0.05, results of the post-hoc Tukey’s multi-comparison test are reported in the graph (* *p* < 0.05); (**B**) Gels porosity determined by gravimetric method, data are reported as mean ± SD (n = 3). One-way ANOVA returned *p* < 0.05, results of the post-hoc Tukey’s multi-comparison test are reported in the graph, (* *p* < 0.05 and ** *p* < 0.01); (**C**) Gels degradation in the presence of lysozyme (full bars), the empty bars represent the relative control gels treated in PBS; data are reported as mean ± SD (n = 3). One-way ANOVA returned *p* < 0.05, results of the post-hoc Tukey’s multi-comparison test are reported in the graph (^#^ *p* < 0.05). A *t*-test was performed against the PBS incubated samples for each gel at each time point; * *p* < 0.05, ** *p* < 0.01 and *** *p* < 0.01; (**D**) Percentage surface calcification of hydrogels incubated at 37°C in SBF for 7, 14 and 21 days as calculated by image analysis via ImageJ and BoneJ from SEM images. Controls were only incubated for few hours in deionized water. Results are reported as mean ± SD (n = 3). Samples labeled with the symbol ^#^ resulted to be significantly different from their control sample (*p* < 0.05). One-way ANOVA returned *p* < 0.05 only when testing CS-HACS at different time points, the results of the post-hoc Tukey’s multi-comparison test are reported in the graph (* *p* < 0.05, ** *p* < 0.01).

Equilibrium swelling provides an idea of the maximum amount of water a hydrogel can absorb. The addition of HACS significantly increased the total amount of water absorbed at equilibrium from 47.5% ± 8.4% to 93.5% ± 34.7% (*p* < 0.05, Figure 3A), likely causes for this could be the significant increase in porosity from 14.5% ± 3.6% to 26.3% ± 1.9% (*p* = 0.01, Figure 3B), the higher interconnectivity within the scaffold and the higher number of available hydrophilic groups or a combination of them.

**Figure 4:**
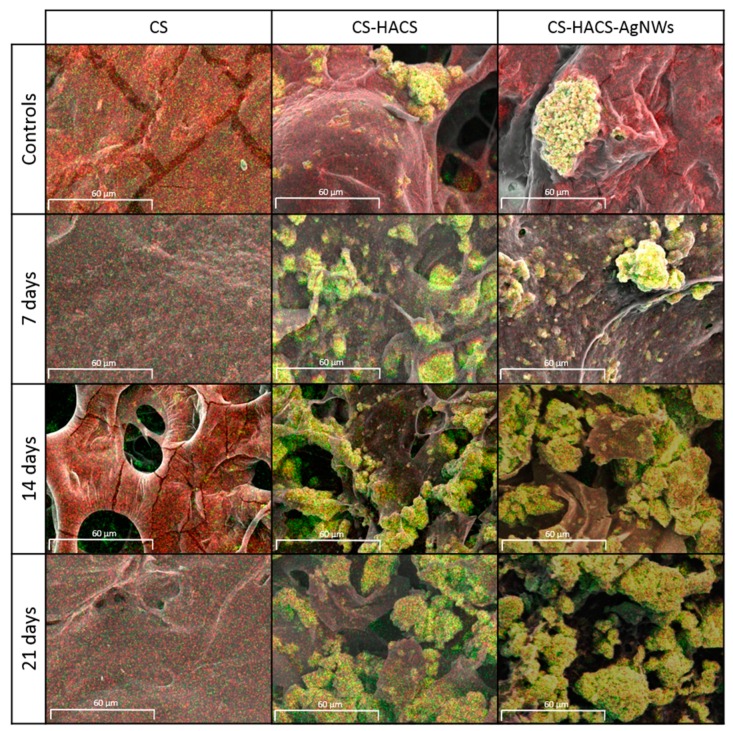
Energy-dispersive X-rays spectroscopy (EDS) coupled SEM images (1000× magnification) of CS, CS-HACS and CS-HACS-AgNWs hydrogels incubated in SBF for 7, 14 and 21 days. Control samples were incubated in water for 5 h. Images show an overlay of SEM pictures with EDS data: Ca (green), P (red) and co-localisation of Ca/P (yellow).

Degradation studies have been performed to understand what factors affect the degradation of the composite hydrogels (see Supplementary Figure S5 online). In the degradation experiment, control hydrogels were incubated in PBS for three weeks and an initial weight loss was observed at seven days. This was then maintained (*p* > 0.05) for the duration of the three weeks, indicating that an initial weight loss due to the solubilization of lightly crosslinked chains is likely to happen in the first week after implantation, however in absence of enzymes, this degradation does not proceed (Figure 3C). A smaller weight loss was observed for CS-HACS (*p* > 0.05) and CS-HACS-AgNWs (*p* < 0.01) at seven days, confirming that enhanced crosslinking is present in these composites. An unchanged residual weight was recorded also for these two composites at two and three weeks. When lysozyme was added, CS gels showed significantly increased degradation during the three weeks with a final total weight loss of 91.7 ± 7.1%. A similar pattern was observed also for the composite gels, for a significant weight loss was observed after three weeks. At all time points, the weight loss of CS-HACS-AgNWs was significantly lower (*p* < 0.01) than both CS and CS-HACS gels. Biomineralization of hydrogels was studied soaking them in SBF for three weeks to evaluate surface Ca/P deposition by SEM-EDS. A significant increase in Ca/P deposit was observed after three weeks of incubation of hydrogels in simulated body fluid (SBF), this was particularly true for CS-HACS hydrogels (Figure 3D and Figure 4). This was expected as the already present hydroxyapatite can function as a nucleation site for the deposition of calcium salts [18]. On the other hand, the absence of hydroxyapatite in CS gels led to a non-significant deposition of salts (*p* > 0.05).

For further investigation of salt deposition within the scaffold, dry scaffolds were scanned by micro-CT with low X-ray energy and low applied voltage (40 kV). As reported by Cancian et al., the region of interest (ROIs) characterized by a higher density may be attributed to salts, such as Ca-P [18]. As expected, quantitative analysis of the hydrogels highlighted a significantly higher salt deposition within the scaffolds containing HACS in comparison to samples not incubated in PBS. Moreover, an increase in salt deposition by time was noticed for both CS-HACS and CS-HACS-AgNWs hydrogels (Supplementary Figures S6 and S7 online).

### 3.3. Ag^+^ Release

**Figure 5:**
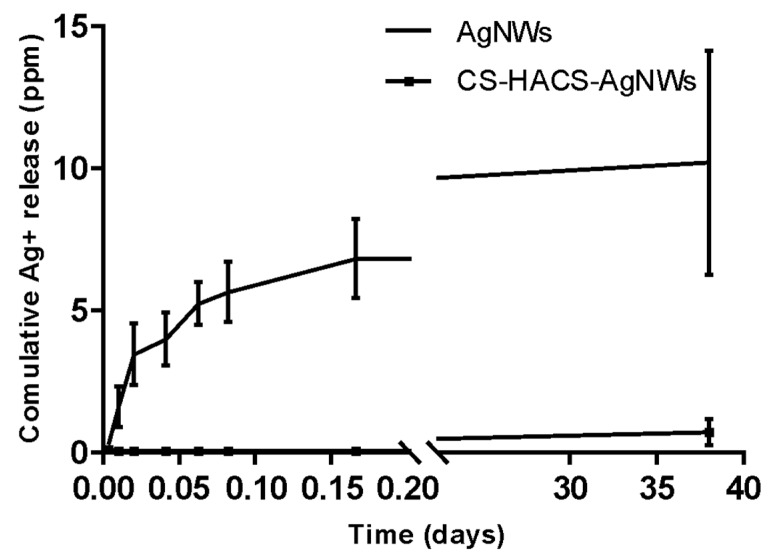
Cumulative release profile of silver cations from AgNWs (triangle) and CS-HACS-AgNWs (square).

AAS was used to determine the total Ag^+^ released from AgNWs and CS-HACS-AgNWs scaffolds. Silver release was studied over a period of 38 days, using ultrapure water as medium. Both materials showed silver release in water (Figure 5), with a faster release at the beginning and a slow sustained release over time. After 38 days, approximately 11 ppm of Ag^+^ were released from AgNWs, while nearly 1 ppm was released from the hydrogels.

### 3.4. Antimicrobial Activity of AgNWs

**Figure 6:**
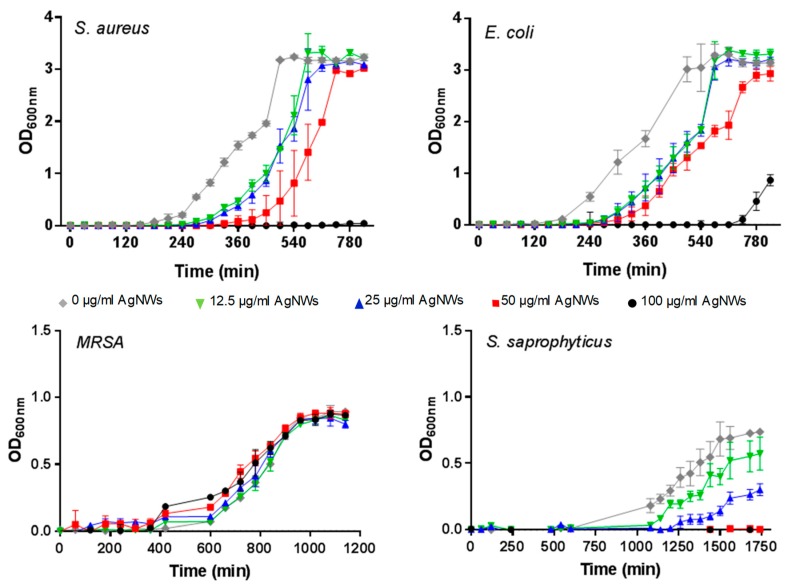
Growth curves of different bacteria in suspension in the presence of 0 (grey), 12.5 (green), 25 (blue), 50 (red) and 100 (black) µg/mL of AgNWs.

The antimicrobial activity of AgNWs was tested against four bacterial strains. Three are gram-positive (*Staphylococcus aureus*, *Staphylococcus saprophyticus* and Methicillin-resistant *Staphylococcus aureus*) while *Escherichia coli* is gram-negative. Staphylococci comprise nearly two-thirds of all pathogens implicated in orthopedic implant infections and thus are clinically relevant in testing materials developed for bone regeneration [19]. Antibacterial efficacy was also assessed against *E. coli* as it often serves as a gram-negative bacterial representative and allowed us to estimate the broadness of the potential antibacterial spectrum. We monitored bacterial growth in liquid culture medium in the presence of increasing concentrations of AgNWs (Figure 6) and determined the lag time before exponential growth (the period when the bacteria are adjusting to the nascent environment), the time required by the bacterial populations to reach stationary phase (when the OD_600nm_ stabilizes due to the rate of cell death equaling the rate of cell division) and the maximum optical density (OD_600nm_) reached at stationary phase (see Supplementary Figure S8 online). *S. aureus* growth was completely inhibited by 100 μg/mL AgNWs (see Supplementary Figure S7 online). The lag phase was found to be statistically increased for the concentration 50 µg/mL (*p* < 0.01) in comparison to the untreated culture. Meanwhile, the time to reach the stationary phase and the maximum OD_600nm_ were not statistically different from the untreated culture (see Supplementary Figure S7 online). For *E. coli* the lag phase was prolonged (compared to the untreated culture) for all the studied concentrations (*p* < 0.001 for 12.5 and 25 µg/mL and *p* < 0.0001 for both 50 and 100 µg/mL; see Supplementary Figure S8 online), with a fourfold increase at 100 µg/mL. MRSA did not show sensitivity to AgNWs at the tested concentrations, while *S. saprophyticus* demonstrated the highest susceptibility, with no growth at all observed when AgNWs were employed at concentrations equal or above 50 µg/mL.

### 3.5. Antibacterial Activity of the Composite Scaffolds

**Figure 7:**
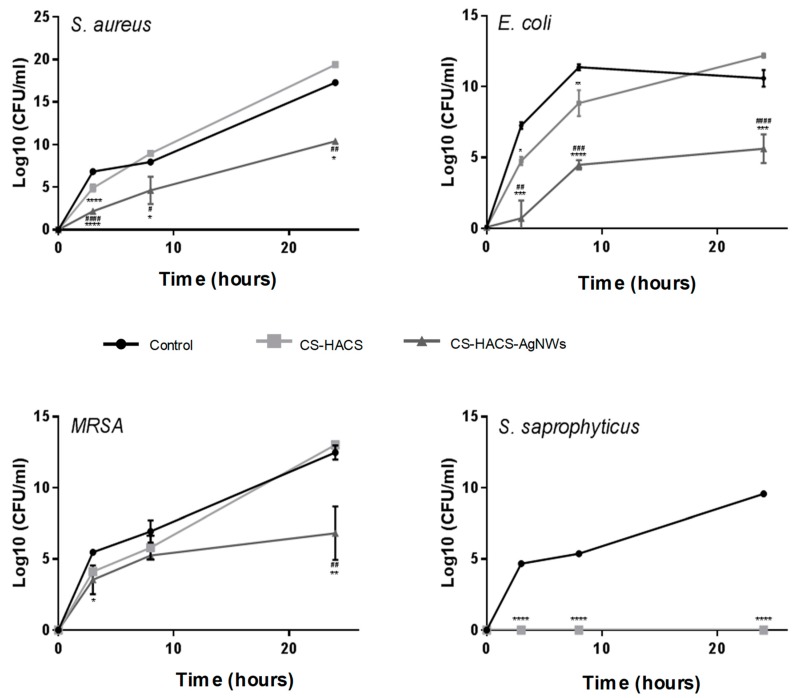
Growth of *S. aureus*, *E. coli*, *MRSA* and *S. saprophyticus* in suspensions containing CS-HACS (light grey), CS-HACS-AgNWs (dark grey) and LB only (black). Results are reported as mean ± SD. One-way ANOVA was performed between different samples, when significant a Tukey’s post-hoc multicomparison test was performed, results are reported on the graph. * represents the statistical difference between scaffolds and bacterial suspensions (* *p* < 0.05, ** *p* < 0.01; *** *p* < 0.001 and **** *p* < 0.0001) while ^#^ represents the statistical difference between CS-HACS and CS-HACS-AgNWs scaffolds (^#^ *p* < 0.05, ^##^ *p* < 0.01; ^###^ *p* < 0.001 and ^####^ *p* < 0.0001).

One of the main objectives of this study was to develop scaffolds imparting antibacterial activity through silver nanowires incorporation and thus we tested the antibacterial activity of CS-HACS-AgNWs hydrogels against CS-HACS scaffolds towards *S. aureus*, *E. coli*, MRSA and *S. saprophyticus*. The analyses were performed incubating the bacterial suspensions with the sterile dried composites. Biofilm formation on CS-HACS and CS-HACS-AgNWs hydrogels was investigated after 24 h, staining the fixed hydrogels to highlight the presence of bacteria. Both of the scaffolds were found to suppress biofilm formation on the scaffolds, as no bacteria have been imaged on the considered surfaces. Hence, both chitosan and silver nanowires appeared to prevent biofilm formation within the considered period of time (see Supplementary Figure S9 online). The antibacterial activity of the composites was studied also in suspension. As reported in Figure 7, CS-HACS-AgNWs scaffolds showed a remarkable bactericidal activity against all the four bacteria strains, giving a growth inhibition of 100% ± 9.6 × 10^−6^ (*p* < 0.001) against *E. coli*, 100% ± 9.2 × 10^−7^ (*p* < 0.000001) against *S. aureus*, 99.99% ± 0.0065 (*p* < 0.05) against MRSA and of 100% ± 0 (*p* < 0.000001) against *S. saprophyticus*, at 24 h. On the other hand, CS-HACS scaffolds did not show the ability to stop the bacterial growth within 24 h, except for *S. saprophyticus*. Overall these results highlighted that the addition of AgNWs was essential in imparting bactericidal activity to hydrogels, and that chitosan and AgNWs might be acting synergistically.

### 3.6. Proliferation and Differentiation of MC3T3-E1

**Figure 8:**
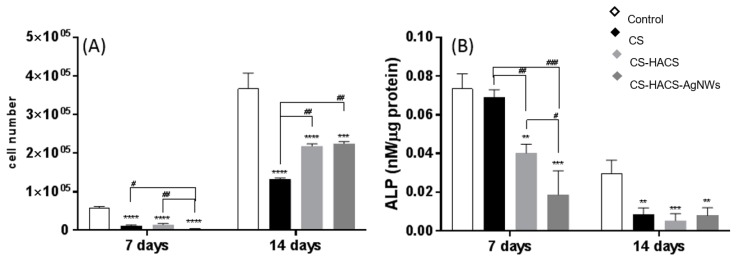
(**A**) ATP produced by MC3T3-E1 cells grown in the presence of the following hydrogels: CS (black), CS-HACS (light grey) and CS-HACS-AgNWs (dark grey). Cultures grown on tissue-culture plates were taken as control (white). Data are reported as mean ± SD (n = 3). One-way ANOVA returned *p* < 0.0001 for 7 and 14 days; (**B**) ALP expression by MC3T3-E1 cells grown in the presence of the same hydrogels. Data reported as mean ± SD (n = 3). One-way ANOVA returned *p* < 0.0001 for 7 days and *p* = 0.0005 for 14 days. The results of the post-hoc Tukey multi comparison test for (**A**,**B**) are reported in the graph: ****, *p* < 0.0001; ***, *p* < 0.001; **, *p* < 0.01 compared to the control; ^#^, *p* < 0.05, ^##^ *p* < 0.001, ^###^ *p* < 0.001.

The biocompatibility of the hydrogels was verified by determining the growth of MC3T3-E1 cells through the adenosine triphosphate (ATP) assay, a well-known reproducible and reliable assay of cell viability (Figure 8A) [29]. The cells responded well to all the hydrogels with an increase in cell number, although they did not proliferate as well as the cells seeded on tissue culture polystyrene plates, taken as control cultures. A significant enhancement of cell growth was detected in HACS containing hydrogels, as shown by the increase in cell number at both days 7 and 14. Although the HACS did not allow the cells to proliferate as well as in the control, it supported cell growth better than the hydrogels containing CS only. At 14 days, no significant difference between the CS-HACS and the CS-HACS-AgNWs was detected. Furthermore, the differentiation of the pre-osteoblast MC3T3-E1 cells was determined in order to evaluate the potential of the hydrogels to support osteoblast differentiation for bone repair. The alkaline phosphatase (ALP) assay was performed as ALP activity is considered to be an early biochemical marker for osteoblast differentiation and an indicator of bone tissue formation [30]. The MC3T3-E1 cells began to differentiate into osteoblasts in all the samples, as confirmed by the presence of ALP activity after seven days in culture (Figure 8B). ALP activity was higher in the control and CS samples than in the other hydrogels. The statistical analysis revealed that there was no statistical difference between the control and CS samples at seven days. With the addition of the hydroxyapatite a decrease in ALP was observed at day 7 while after 14 days no difference between CS and CS-HACS gels was detected. At day 14, a decrease in enzyme activity was visible in all cultures; this is expected as the highest concentration of ALP is expressed during the early stages of bone formation [31].

## 4. Discussion

Bone fractures are often exacerbated by the occurrence of bacterial infections that can lead to prolonged or even absent bone healing. Therefore, 3D-engineered scaffolds with both bioactive and infection control capabilities are urgently needed. In this study, we aimed to develop the first biodegradable and bioactive injectable hydrogel enriched with AgNWs with intrinsic antibacterial properties. Injectable hydrogels are potentially ideal pharmaceutical formulations for bone regeneration as they reduce the discomfort of the patient due to their non-invasive administration. However, a relatively fast gelation time, once administrated, is essential for this type of formulation as this allows an effective entrapment of additives or cells at the site of application. CS-GP hydrogels are known for being thermosensitive as they undergo solution-gel transition in a temperature-dependent manner [32]. Our in vitro studies demonstrated that addition of HACS significantly diminished gelation time at body temperature, in comparison to CS only gels, whereas AgNWs did not affect this property. This behavior may be attributed to the higher number of functional groups available for ionic interactions in CS-HACS than in CS hydrogels. Even though chitosan-based hydrogels are ideal scaffolds for tissue regeneration as they mimic the extracellular matrix, they present inadequate mechanical performance, which makes them too weak for applications in the musculoskeletal system. Multiple approaches have been taken by researchers to overcome this problem, like incorporating nanoparticles [16]. In this study, the addition of HACS significantly enhanced the compressive gel strength in comparison to the chitosan only gel. This result was further improved by the addition of AgNWs, as a consequence of their metallic nature—which provides high strength—and the one-dimensional morphology that defines the mechanical behavior. In fact, Cao et al. showed that the smaller the diameter the stronger AgNWs are; the AgNWs used in this study, with a diameter around 99 nm, present a behavior between brittle and ductile [33]. A further important property of a hydrogel for tissue regeneration is its swelling capacity: swelling is related to water uptake from the surrounding tissues that favors nutrient and cell migration [34,35]. Chitosan is known for its hydrophilic nature due to the presence of several hydroxyl and amino groups able to form hydrogen bonding with the surrounding water. The inclusion of HACS into CS-based hydrogels ensured a higher water uptake, due to the higher number of hydrophilic groups but also to the higher porosity of these gels in comparison to the CS only gels. Thus, HACS is not only acting as a crosslinker, as shown by the enhanced mechanical behavior, but also as a spacer between polymeric chains enhancing the porosity and hydrophilicity of the composites. During bone repair, the aim is to employ materials that will be resorbed while the new bone is forming and will allow drug release. Thus, degradation studies have been performed to understand what factors affect the resorption of the composite hydrogels. The in vitro degradation studies were carried out in the presence of lysozyme, an enzyme that degrades chitosan in vivo. Based on gravimetric analysis and observation by SEM, CS-HACS-AgNWs hydrogels showed significantly reduced degradation in comparison to the chitosan only gel. The addition of HACS composites had no effect on the degradation in comparison to chitosan hydrogels, in good agreement with the findings reported by Dhivya et al. [36]. This suggests that the introduction of AgNWs might be used to regulate and delay the degradation of the gels. A further important step for bone regeneration is mineralization of the tissue. The in vitro apatite formation ability of a scaffold can be correlated to its in vivo bone-bonding ability and capacity to absorb calcium and phosphate ions from the surrounding body fluids. Indeed, Ca and P are used by the human body to form hydroxyapatite, the bone mineral that constitutes nearly the 65% of bone weight. Thus, the presence of hydroxyapatite on the surface of the scaffolds is essential to promote the nucleation and growth of calcium phosphate. Our in vitro study showed that more Ca/P deposits increased over time on the surface of the hydrogels if hydroxyapatite was present in the composite. The data presented support the key role played by hydroxyapatite in the bioactivity of the composite hydrogels and at the same time demonstrate that the addition of AgNWs further supports the bioactivity. As observed previously for carbon nanotubes, AgNWs also seem to favor a homogeneous salt deposition [18]. As mentioned, bacterial infections are a burden after bone fracture surgeries. The controlled release of silver cation is a key factor for preventing and treating infections. Indeed, Ag^+^ can interact with electron donor groups in biological molecules containing sulphur, oxygen or nitrogen, causing damage to the microorganisms [2]. Silver is present in AgNWs in the metallic state, this has no antibacterial properties, however it is able to react with moisture and O_2_ in the culture medium, producing ionized Ag^+^. This is highly reactive as it directly interacts with proteins and promotes structural changes in the bacterial cell wall, leading to cell distortion and death. As reported by Kumar et al., the steady and prolonged release of silver from scaffolds at a minimum concentration level of 0.1 part per billion (ppb) can provide effective antimicrobial activity [37]. In our study we have explored the antibacterial activity of AgNWs and CS-HACS-AgNWs. The cumulative release profiles of silver cations from AgNWs and hydrogels indicate that the total silver ion released is well within the potential toxic limit mentioned for human cells which is 10 ppm (µg/mL), but high enough to have the potential to inhibit bacterial growth. The antibacterial properties of AgNWs against suspension cultures of *E. coli*, *S. aureus*, MRSA and *S. saprophyticus* were evaluated. AgNWs were able to prevent the growth of *S. aureus* and *S. saprophyticus* at concentrations higher than 50 µg/mL, they affected the lag phase of *S. aureus* at concentrations higher than 50 µg/mL and of *E. coli* for all the studied concentrations. Moreover, the OD_600_ of *E. coli* stationary phase was statistically different for the concentration 50 µg/mL. Our results are in good agreement with Cui and Liu [38]: they carried out a similar experiment for *E. coli* showing that AgNWs were able to affect the lag phase for concentrations equal or higher than 12.5 µg/mL. However, in their work, concentrations of AgNWs equal to 50 µg/mL were able to prevent bacterial growth. In agreement with our study, Hong et al. reported a 6 h delay of *E. coli* growth for the AgNWs concentration 50 µg/mL, without any inhibition [39]. Finally, in our work, *E. coli* was found to exhibit greater sensitivity to AgNWs than *S. aureus*; this trend has been reported for silver nanoparticles too [40]. Unfortunately, no activity was observed against MRSA. The antimicrobial properties of the composite hydrogels were evaluated both in suspension and on the scaffold. On one hand, chitosan is known to exhibit antimicrobial properties against fungi, yeasts, viruses and bacteria, including *E. coli* and *S. aureus*, inhibiting DNA transcription and mRNA synthesis [41]. On the other, the introduction of AgNWs was expected to enhance these antibacterial properties due to the release of Ag^+^ from the scaffolds. Indeed, CS-HACS-AgNWs could reduce the bacterial growth of over 99% for all the studied species over a period of 24 h in suspension, in comparison to CS-HACS hydrogels. Moreover, biofilm formation was prevented on the hydrogels with or without AgNWs, over a period of 24 h. The ability of CS-HACS hydrogels to inhibit bacterial growth on the scaffold, may be explained by the direct interaction of positively charged chitosan with negatively charged bacterial cell surface, which caused cell membrane disruption leading to death [42]. These results are in good agreement with previous literature studies, regarding the ability of low viscosity chitosan to prevent *Staphylococcus* and *E. coli* growth [43,44]. The most interesting results were obtained when the scaffolds were tested against MRSA. AgNWs alone were not able to exert any effect on MRSA at concentrations as high as 100 µg/mL, but a very high activity was observed when the nanowires were included in the scaffold, even though the release studies suggested that in this case bacteria will be subjected to much lower concentrations of silver ions. The combination of chitosan and AgNWs acted in a synergistic manner, with chitosan increasing the bacterial cell wall permeability and allowing an easier access of Ag^+^ into the cell [45]. A further important property of a scaffold for bone tissue engineering is osteogenicity. Researchers define as osteogenic a scaffold that favors the adhesion and proliferation of osteoprogenitor cells, therefore the proliferation of pre-osteoblasts (MC3T3-E1) was studied. Cell number significantly increased on CS-HACS and CS-HACS-AgNWs more than in gels with CS only over a period of 14 days. This indicates a good biocompatibility of the studied biomaterials. Cell differentiation into osteoblasts was investigated by ALP assay that is a marker of the early stages of osteogenic differentiation. ALP plays a role in the conversion of inorganic pyrophosphate into inorganic phosphate, a process that promotes the mineralization of the deposited bone matrix [46]. Over a period of 14 days, all the studied hydrogels promoted cell differentiation at the same level. However, there was a decrease for all the studied cultures between 7 and 14 days. ALP decrease by time might be explained due to the cells entering a new development stage and not because the hydrogels are unable to sustain osteogenic differentiation, however this should be further investigated. Further investigations are also required to understand the effect of AgNWs on the production and detection of ALP at 7 days. It is known that silver has a high affinity for thiol groups, it is therefore possible to postulate the formation of a complex between silver and ALP that might be responsible for the modulation of the enzyme activity as seen in vivo by Adeyemi and Adewumi [47].

## 5. Conclusions

The addition of silver nanowires into the chitosan-hydroxyapatite hydrogel scaffolds did not interfere with the rapid sol/gel transition at body temperature, enhancing both the mechanical and the bioactive properties of the materials. The presence of AgNWs induced a reduction in the degradation rate of the hydrogels, suggesting that composites with a tailored degradation pattern can be designed in this manner. The presence of chitosan in the scaffolds allowed for a slow controlled release of Ag^+^ and provided a synergistic effect towards antibacterial activity, manifested also against resistant bacterial strains such as MRSA. Furthermore, the gels proved to be biocompatible and bioactive, allowing in vitro cell proliferation. The results obtained warrant further investigations into clinical application, as the formulation is injectable and has proven bioactive, antimicrobial and biocompatible properties. Further, it can be used as a controlled delivery platform not only for Ag^+^ but potentially for other therapeutic molecules.

## Figures and Tables

**Table 1:** Components used in the formulation of each millilitre of hydrogel. CS: chitosan; HACS: hydroxyapatite chitosan composite; AgNWs: silver nanowires; GP: glycerol phosphate.

Component	CS	CS-HACS	CS-HACS-AgNWs	CS-AgNWs
Lactic acid (0.1 M)	0.9 mL	0.9 mL	0.9 mL	0.9 mL
Chitosan	20 mg	20 mg	20 mg	20 mg
HACS	--	8.6 mg	8.6 mg	--
AgNWs (powder)	--	--	4 mg	4 mg
GP (1.12 g/mL)	0.1 mL	0.1 mL	0.1 mL	0.1 mL

## Supplementary Materials

The following are available online at https://www.mdpi.com/1999-4923/11/3/116/s1, Figure S1. AgNWs size distribution obtained by SEM image analysis. (A) Length (mean 5.03 ± 1.85 µm; n = 28) and (B) diameter (mean 99.45 ± 20.20 nm, n = 28). Shapiro-Wilk normality test returned *p* > 0.05 for both length and diameter, Figure S2. SEM images of HACS (top) and FTIR spectra of the three hydrogels (bottom). Figure S3. Physical characterisation of the hydrogels: CS (black), CS-HACS-AgNWs (black background with dark grey lines), CS-HACS (light grey) and CS-HACS-AgNWs (dark grey). Gelation time calculated by the inverted tube method. Data are reported as mean ± SD (n = 9). One-way ANOVA returned *p* < 0.0001. Post-hoc Tukey’s comparison test results are shown in the graph: **** indicates *p* < 0.0001 and ** indicates *p* < 0.001., Figure S4. Gel strength calculated by texture analysis before (empty bars) and after (filled bars) gelation. Data are represented as mean ± SD (n = 3). The *t*-test performed on all samples before and after gelation showed significantly different strength values for all gels (^$$^ indicates *p* < 0.01 and ^$$$$^ *p* < 0.0001). One-way ANOVA to compare the different formulations returned *p* < 0.01 before and *p* < 0.0001 after gelation. Post-hoc Tukey’s test results showed that before gelation only CS is different from all other gels (^#^ *p* < 0.05), while the individual results for after gelation are reported on the graph (** *p* < 0.01; **** *p* < 0.0001)., Figure S5. SEM images (200× magnification) of the degradation process in PBS and in lysozyme for CS, CS-HACS and CS-HACS-AgNWs gels at 7, 14 and 21 days., Figure S6. MicroCT images of CS, CS-HACS and CS-HACS-AgNWs hydrogels incubated in SBF for 7, 14 and 21 days. The arrow indicates an area of mineralized tissue characterized by a higher X-ray absorption than the surrounding hydrogel., Figure S7. Average % salt deposition on different types of hydrogel samples (from microCT). Data are reported as mean ± SD (n = 3). One-way ANOVA returned *p* < 0.05 for both gels containing AgNWs, compared to control, and *p* < 0.001 was obtained for CS-HACS hydrogels both at 14 and 21 days compared to the control. A Tukey’s post-hoc multicomparison test was performed, results are reported on the graph. The symbol* indicates statistical difference when comparing the two gels containing HACS at each time point. The symbol ^#^ indicates statistical difference of a gel in comparison to the chitosan control at a set time point. The symbol ^$^ is used for specific comparisons as indicated in the graph., Figure S8. Bacterial growth lag time, time to reach the stationary phase, and O.D. at stationary phase for different bacteria in suspension treated with 0 (grey), 12.5 (green), 25 (blue), 50 (red) and 100 (black) µg/mL of AgNWs. Data are reported as mean ± S.D. (n = 3). One-way ANOVA was performed between different AgNWs concentrations against the same bacteria, when significant a Tukey’s post-hoc multicomparison test was performed, results are reported on the graph. The symbol* indicates statistical difference when compared to 0 µg/mL, the symbol ^#^ indicates comparison with 100 µg/mL, the symbol ^$^ is used for specific comparisons as indicated in the graph., Figure S9. Microscopy images of biofilm formation on CS-HACS, CS-HACS-AgNWs hydrogels and controls after fuchsin (*E. coli*) or crystal violet (*S. aureus*, MRSA and *S. saprophyticus*) staining. Magnification 100×.

## References

[1] Pokropivny V.V., Skorokhod V.V. (2008). New dimensionality classifications of nanostructures. Phys. E Low-Dimens. Syst. Nanostruct..

[2] Jones R., Draheim R., Roldo M. (2018). Silver Nanowires: Synthesis, Antibacterial Activity and Biomedical Applications. Appl. Sci..

[3] Zhang P., Wyman I., Hu J., Lin S., Zhong Z., Tu Y., Huang Z., Wei Y. (2017). Silver nanowires: Synthesis technologies, growth mechanism and multifunctional applications. Mater. Sci. Eng. B.

[4] Majeed Khan M.A., Kumar S., Ahamed M., Alrokayan S.A., AlSalhi M.S. (2011). Structural and thermal studies of silver nanoparticles and electrical transport study of their thin films. Nanoscale Res. Lett..

[5] Hsueh Y.-H., Lin K.-S., Ke W.-J., Hsieh C.-T., Chiang C.-L., Tzou D.-Y., Liu S.-T. (2015). The Antimicrobial Properties of Silver Nanoparticles in Bacillus subtilis Are Mediated by Released Ag+ Ions. PLoS ONE.

[6] Hebeish A., El-Rafie M.H., EL-Sheikh M.A., Seleem A.A., El-Naggar M.E. (2014). Antimicrobial wound dressing and anti-inflammatory efficacy of silver nanoparticles. Int. J. Biol. Macromol..

[7] Roe D., Karandikar B., Bonn-Savage N., Gibbins B., Roullet J.-B. (2008). Antimicrobial surface functionalization of plastic catheters by silver nanoparticles. J. Antimicrob. Chemother..

[8] Andara M., Agarwal A., Scholvin D., Gerhardt R.A., Doraiswamy A., Jin C., Narayan R.J., Shih C.-C., Shih C.-M., Lin S.-J. (2006). Hemocompatibility of diamondlike carbon–metal composite thin films. Diam. Relat. Mater..

[9] Alt V., Bechert T., Steinrücke P., Wagener M., Seidel P., Dingeldein E., Domann E., Schnettler R. (2004). An in vitro assessment of the antibacterial properties and cytotoxicity of nanoparticulate silver bone cement. Biomaterials.

[10] Yoshida K., Tanagawa M., Matsumoto S., Yamada T., Atsuta M. (1999). Antibacterial activity of resin composites with silver-containing materials. Eur. J. Oral Sci..

[11] Nateghi M.R., Shateri-Khalilabad M. (2015). Silver nanowire-functionalized cotton fabric. Carbohydr. Polym..

[12] Shahzadi K., Wu L., Ge X., Zhao F., Li H., Pang S., Jiang Y., Guan J., Mu X. (2016). Preparation and characterization of bio-based hybrid film containing chitosan and silver nanowires. Carbohydr. Polym..

[13] Wickham A., Vagin M., Khalaf H., Bertazzo S., Hodder P., Dånmark S., Bengtsson T., Altimiras J., Aili D. (2016). Electroactive Biomimetic Collagen-Silver Nanowire Composite Scaffolds. Nanoscale.

[14] Pang S., Ding L., Chen X., Xing M., Sun Z., Tao J. (2018). Preparation and Properties of Electro-Spun PVP / Silver Nanowire Composite Nanofibers. Compos. Mater..

[15] Killion J.A., Kehoe S., Geever L.M., Devine D.M., Sheehan E., Boyd D., Higginbotham C.L. (2013). Hydrogel/bioactive glass composites for bone regeneration applications: Synthesis and characterisation. Mater. Sci. Eng. C.

[16] Tozzi G., De Mori A., Oliveira A., Roldo M. (2016). Composite hydrogels for bone regeneration. Materials.

[17] Yasmeen S., Lo M.K., Bajracharya S., Roldo M. (2014). Injectable scaffolds for bone regeneration. Langmuir.

[18] Cancian G., Tozzi G., Hussain A.A., De Mori A., Roldo M. (2016). Carbon nanotubes play an important role in the spatial arrangement of calcium deposits in hydrogels for bone regeneration. J. Mater. Sci. Mater. Med..

[19] Ribeiro M., Monteiro F.J., Ferraz M.P. (2012). Infection of orthopedic implants with emphasis on bacterial adhesion process and techniques used in studying bacterial-material interactions. Biomatter.

[20] Nair M.B., Kretlow J.D., Mikos A.G., Kasper F.K. (2011). Infection and tissue engineering in segmental bone defects—A mini review. Curr. Opin. Biotechnol..

[21] Yang C., Tang Y., Su Z., Zhang Z., Fang C. (2015). Preparation of Silver Nanowires via a Rapid, Scalable and Green Pathway. J. Mater. Sci. Technol..

[22] Ganji F., Abdekhodaie M.J., Ramanzi A. (2007). Gelation time and degradation rate of chitosan-based injectable hydrogel. J. Sol-Gel Sci. Technol..

[23] Nanda S., Sood N., Reddy B.V.K., Markandeywar T.S. (2013). Preparation and Characterization of Poly (vinyl alcohol)-chondroitin Sulphate Hydrogel as Scaffolds for Articular Cartilage Regeneration. Indian J. Mater. Sci..

[24] Kokubo T., Takadama H. (2006). How useful is SBF in predicting in vivo bone bioactivity?. Biomaterials.

[25] Xiong Y., Xie Y., Wu C., Yang J., Li Z., Xu F. (2003). Formation of Silver Nanowires Through a Sandwiched Reduction Process. Adv. Mater..

[26] Bari B., Lee J., Jang T., Won P., Ko S.H., Alamgir K., Arshad M., Guo L.J. (2016). Simple hydrothermal synthesis of very-long and thin silver nanowires and their application in high quality transparent electrodes. J. Mater. Chem. A.

[27] Lin J.-Y., Hsueh Y.-L., Huang J.-J. (2014). The concentration effect of capping agent for synthesis of silver nanowire by using the polyol method. J. Solid State Chem..

[28] Moreno I., Navascues N., Arruebo M., Irusta S., Santamaria J. (2013). Facile preparation of transparent and conductive polymer films based on silver nanowire/polycarbonate nanocomposites. Nanotechnology.

[29] Petty R.D., Sutherland L.A., Hunter E.M., Cree I.A. (1995). Comparison of MTT and ATP-based assays for the measurement of viable cell number. J. Biol. Chem..

[30] Narang A.S., Delmarre D., Gao D. (2007). Stable drug encapsulation in micelles and microemulsions. Int. J. Pharm..

[31] Paganelli G., Magnani P., Zito F., Villa E., Sudati F., Lopalco L., Rossetti C., Malcovati M., Chiolerio F., Seccamani E. (1991). Three-step monoclonal anitibody tumor targeting in carcinoembryonic abtigen-posiive patients. Cancer Res..

[32] Ahmadi R., de Bruijn J.D. (2008). Biocompatibility and gelation of chitosan–glycerol phosphate hydrogels. J. Biomed. Mater. Res. Part A.

[33] Cao K., Han Y., Zhang H., Gao L., Yang H. (2018). Size-dependent fracture behavior of silver nanowires. Nanothechnology.

[34] Gnanaprakasam Thankam F., Muthu J. (2013). Influence of plasma protein-hydrogel interaction moderated by absorption of water on long-term cell viability in amphiphilic biosynthetic hydrogels. RSC Adv..

[35] Seyednejad H., Gawlitta D., Kuiper R.V., de Bruin A., van Nostrum C.F., Vermonden T., Dhert W.J.A., Hennink W.E. (2012). In vivo biocompatibility and biodegradation of 3D-printed porous scaffolds based on a hydroxyl-functionalized poly(ε-caprolactone). Biomaterials.

[36] Dhivya S., Saravanan S., Sastry T.P., Selvamurugan N. (2015). Nanohydroxyapatite-reinforced chitosan composite hydrogel for bone tissue repair in vitro and in vivo. J. Nanobiotechnol..

[37] Kumar R., Münstedt H. (2005). Silver ion release from antimicrobial polyamide/silver composites. Biomaterials.

[38] Li Z., Cen L., Zhao L., Cui L., Liu W., Cao Y. (2010). Preparation and evaluation of thiolated chitosan scaffolds for tissue engineering. J. Biomed. Mater. Res. Part A.

[39] Hong X., Wen J., Xiong X., Hu Y. (2016). Shape effect on the antibacterial activity of silver nanoparticles synthesized via a microwave-assisted method. Environ. Sci. Pollut. Res..

[40] Dhand V., Soumya L., Bharadwaj S., Chakra S., Bhatt D., Sreedhar B. (2016). Green synthesis of silver nanoparticles using Coffea arabica seed extract and its antibacterial activity. Mater. Sci. Eng. C.

[41] Cheung R.C.F., Ng T.B., Wong J.H., Chan W.Y. (2015). Chitosan: An Update on Potential Biomedical and Pharmaceutical Applications. Mar. Drugs.

[42] Venkatesan J., Jayakumar R., Mohandas A., Bhatnagar I., Kim S.-K. (2014). Antimicrobial Activity of Chitosan-Carbon Nanotube Hydrogels. Materials.

[43] Asli A., Brouillette E., Ster C., Ghinet M.G., Brzezinski R., Lacasse P., Jacques M., Malouin F. (2017). Antibiofilm and antibacterial effects of specific chitosan molecules on Staphylococcus aureus isolates associated with bovine mastitis. PLoS ONE.

[44] Goy R.C., Morais S.T.B., Assis O.B.G. (2016). Evaluation of the antimicrobial activity of chitosan and its quaternized derivative on E. coli and S. aureus growth. Rev. Bras. Farm..

[45] Huang L., Dai T., Xuan Y., Tegos G.P., Hamblin M.R. (2011). Synergistic Combination of Chitosan Acetate with Nanoparticle Silver as a Topical Antimicrobial: Efficacy against Bacterial Burn Infections. Antimicrob. Agents Chemother..

[46] Pujari-Palmer M., Pujari-Palmer S., Lu X., Lind T., Melhus H., Engstrand T., Karlsson-Ott M., Engqvist H. (2016). Pyrophosphate Stimulates Differentiation, Matrix Gene Expression and Alkaline Phosphatase Activity in Osteoblasts. PLoS ONE.

[47] Adeyemi O.S., Adewumi I. (2014). Biochemical Evaluation of Silver Nanoparticles in Wistar Rats. Int. Sch. Res. Not..

