# Supplementary material for: Sustained Release from Injectable Composite Gels Loaded with Silver Nanowires Designed to Combat Bacterial Resistance in Bone Regeneration Applications
Source: Pharmaceutics. 2019 Mar 12;11(3):116. doi: 10.3390/pharmaceutics11030116 (PMC6471462; doi:10.3390/pharmaceutics11030116)
Supplement: Supplementary file 1 [file pharmaceutics-11-00116-s001.pdf]

Sustained release from injectable composite gels loaded with silver nanowires designed to combat bacterial resistance in bone regeneration applications

A. De Mori, M. Hafidh, N. Mele, R. Yusuf, G. Cerri, E. Gavini, G. Tozzi, E. Barbu, M. T. Conconi, R. Draheim and M. Roldo

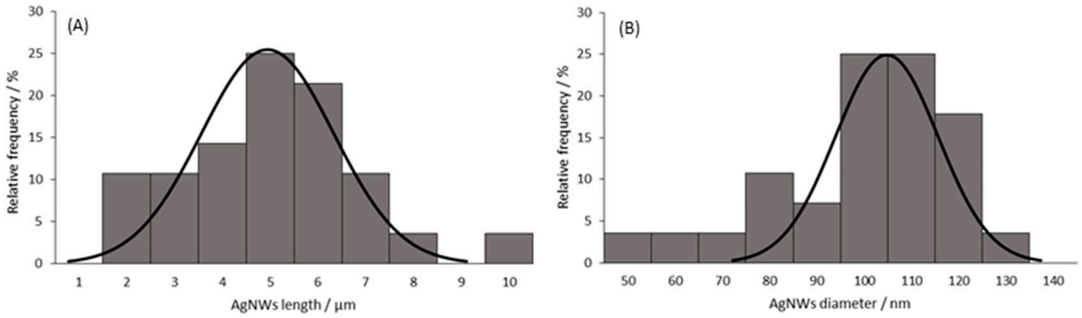

Figure S1. AgNWs size distribution obtained by SEM image analysis. (A) Length (mean 5.03 ± 1.85 µm; n = 28) and (B) diameter (mean 99.45 ± 20.20 nm, n = 28). Shapiro-Wilk normality test returned $p > 0.05$ for both length and diameter.

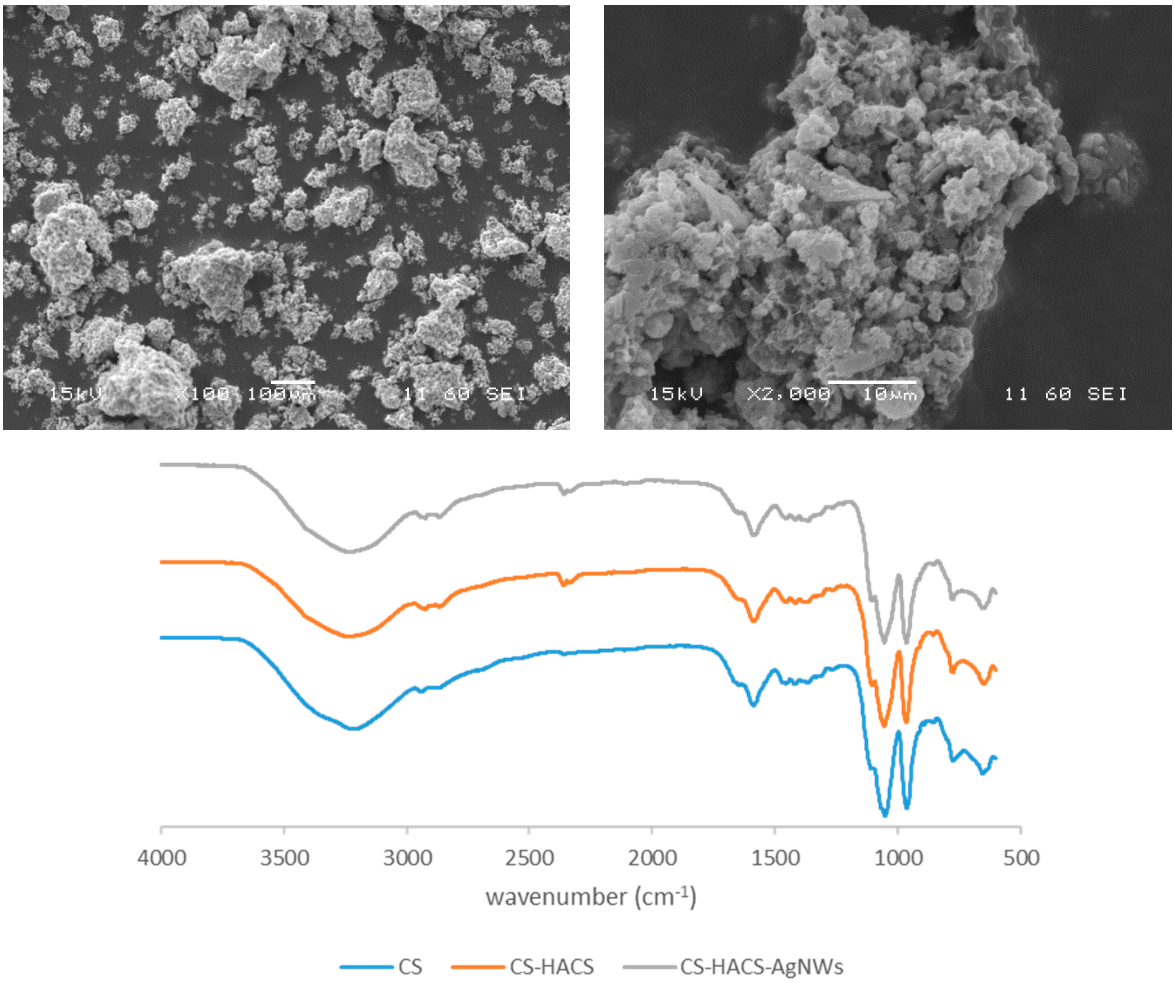

Figure S2. SEM images of HACS (top) and FTIR spectra of the three hydrogels (bottom).

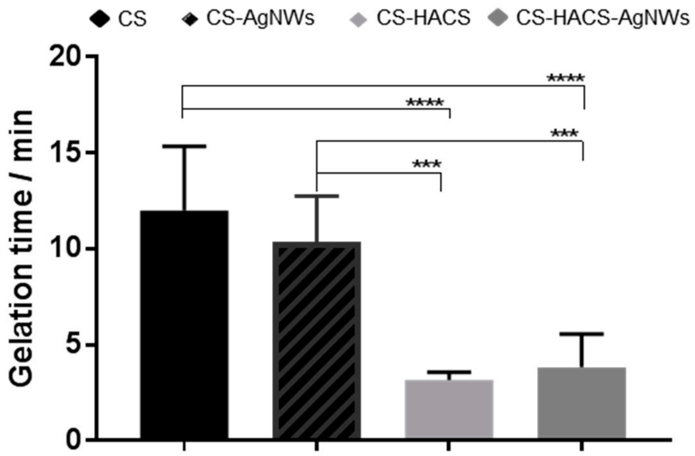

Figure S3. Physical characterisation of the hydrogels: CS (black), CS-HACS-AgNWs (black background with dark grey lines), CS-HACS (light grey) and CS-HACS-AgNWs (dark grey). Gelation time calculated by the inverted tube method. Data are reported as mean ± SD (n = 9). One-way ANOVA returned $p < 0.0001$. Post-hoc Tukey's comparison test results are shown in the graph: **** indicates $p < 0.0001$ and ** indicates $p < 0.001$.

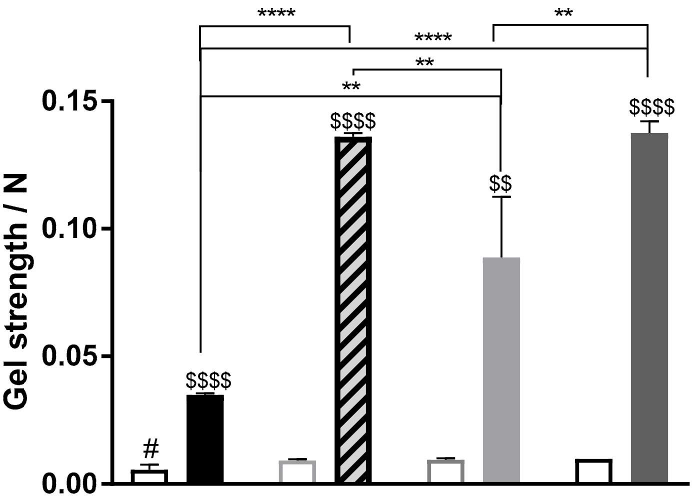

Figure S4. Gel strength calculated by texture analysis before (empty bars) and after (filled bars) gelation. Data are represented as mean ± SD (n = 3). The t-test performed on all samples before and after gelation showed significantly different strength values for all gels ($$ indicates $p < 0.01$ and $$$$ $p < 0.0001$). One-way ANOVA to compare the different formulations returned $p < 0.01$ before and $p < 0.0001$ after gelation. Post-hoc Tukey's test results showed that before gelation only CS is different from all other gels (# $p < 0.05$), while the individual results for after gelation are reported on the graph (** $p < 0.01$; **** $p < 0.0001$).

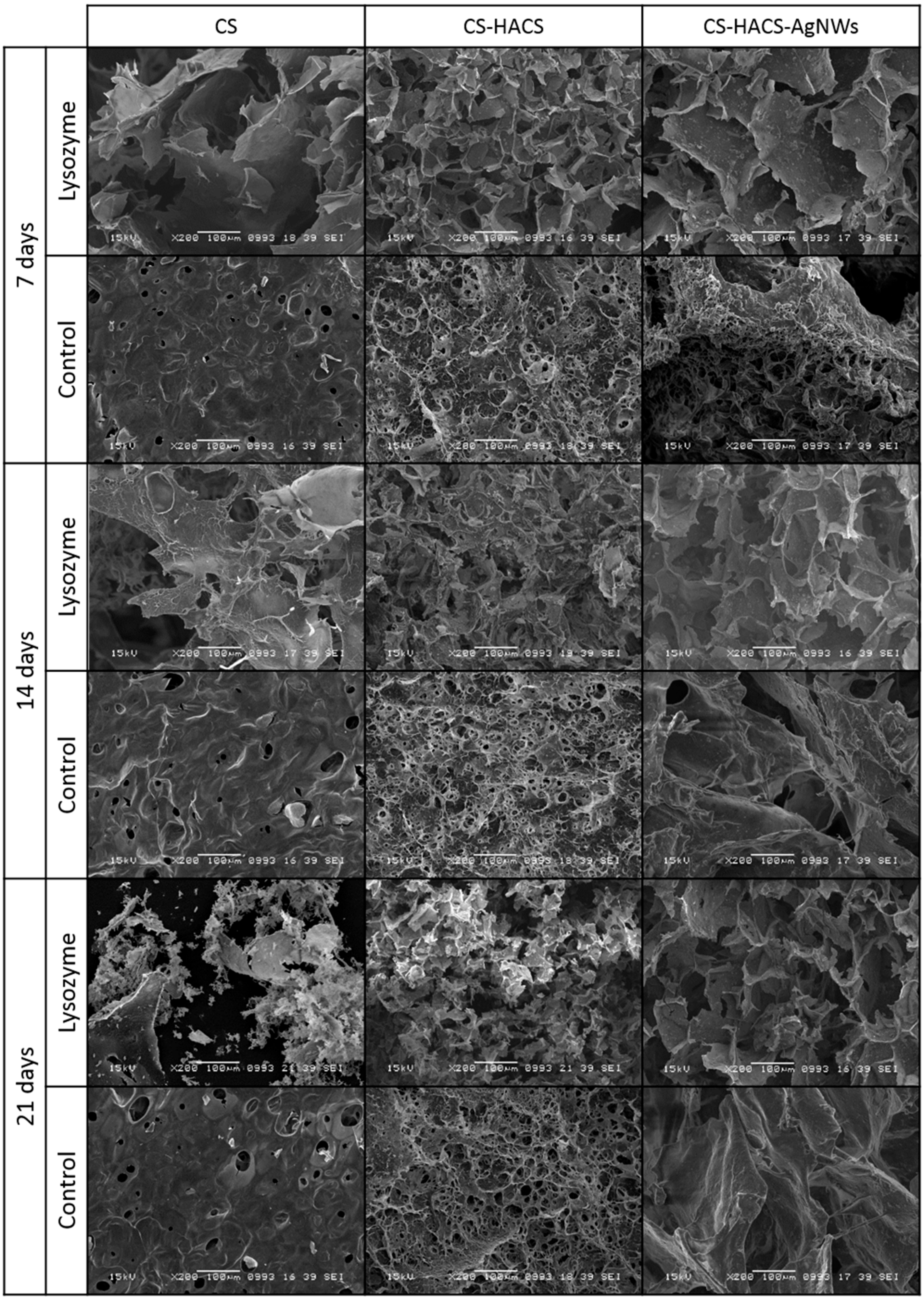

Figure S5. SEM images (200× magnification) of the degradation process in PBS and in lysozyme for CS, CS-HACS and CS-HACS-AgNWs gels at 7, 14 and 21 days.

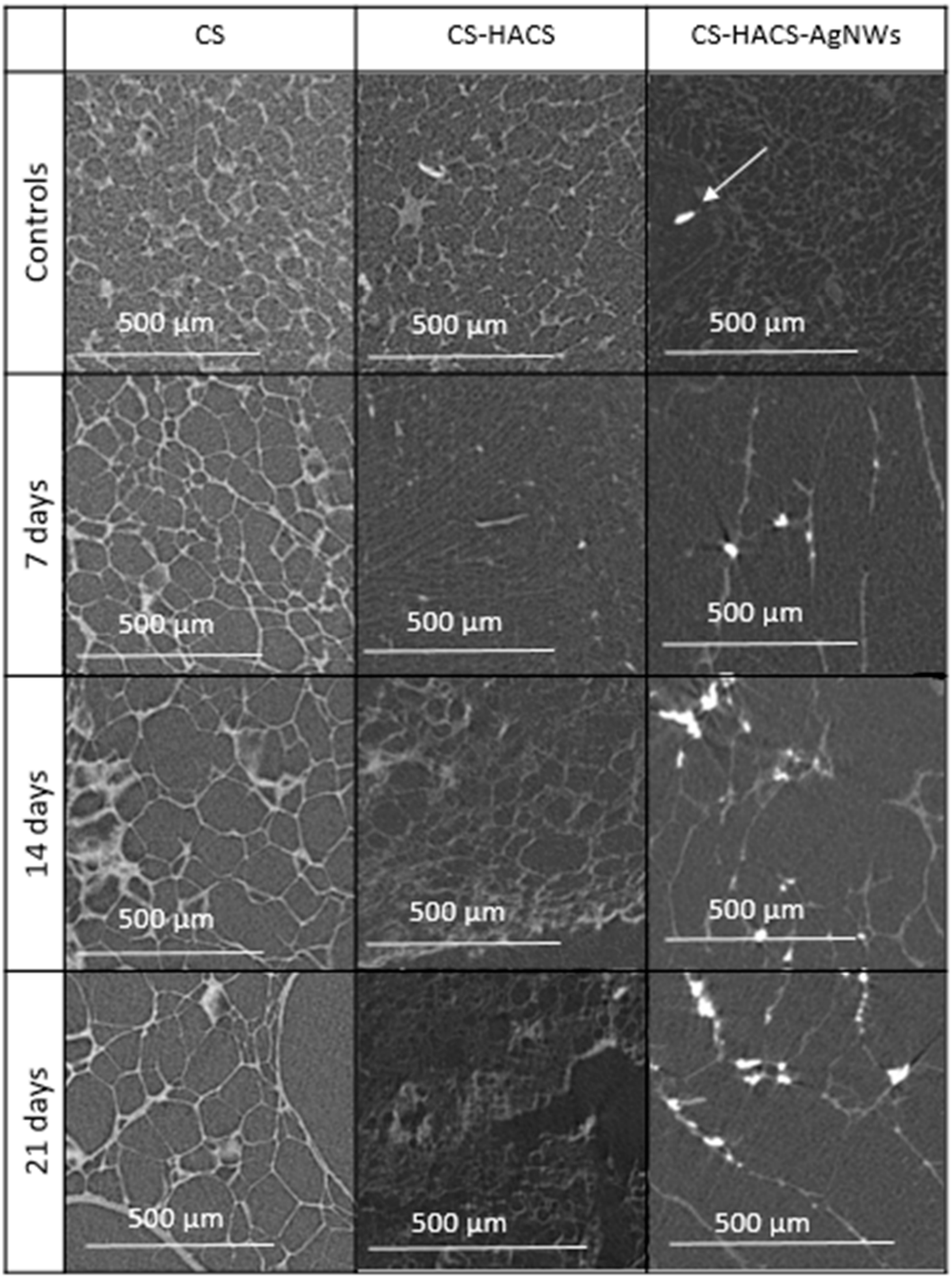

Figure S6. MicroCT images of CS, CS-HACS and CS-HACS-AgNWs hydrogels incubated in SBF for 7, 14 and 21 days. The arrow indicates an area of mineralized tissue characterized by a higher X-ray absorption than the surrounding hydrogel.

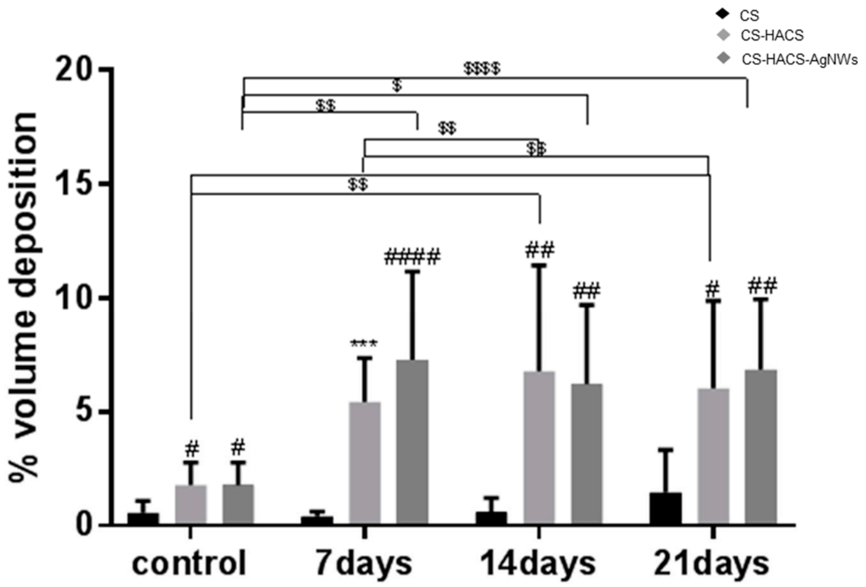

Figure S7. Average % salt deposition on different types of hydrogel samples (from microCT). Data are reported as mean ± SD (n = 3). One-way ANOVA returned $p < 0.05$ for both gels containing AgNWs, compared to control, and $p < 0.001$ was obtained for CS-HACS hydrogels both at 14 and 21 days compared to the control. A Tukey's post-hoc multicomparison test was performed, results are reported on the graph. The symbol* indicates statistical difference when comparing the two gels containing HACS at each time point. The symbol # indicates statistical difference of a gel in comparison to the chitosan control at a set time point. The symbol $ is used for specific comparisons as indicated in the graph.

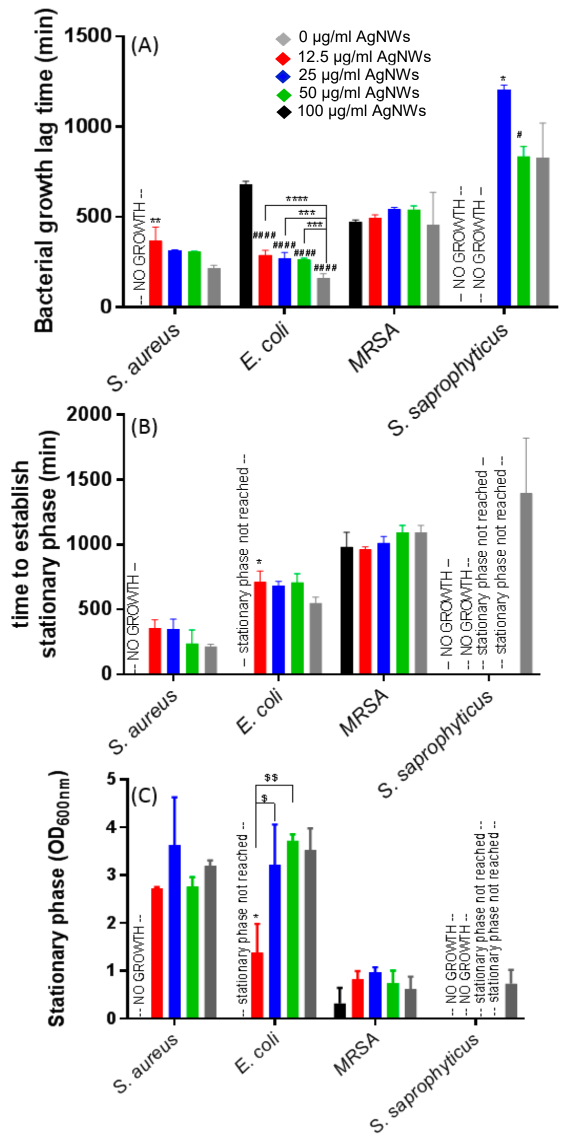

Figure S8. Bacterial growth lag time, time to reach the stationary phase, and O.D. at stationary phase for different bacteria in suspension treated with 0 (grey), 12.5 (green), 25 (blue), 50 (red) and 100 (black) µg/ml of AgNWs. Data are reported as mean ± S.D. (n=3). One-way ANOVA was performed between different AgNWs concentrations against the same bacteria, when significant a Tukey's post-hoc multi-comparison test was performed, results are reported on the graph. The symbol* indicates statistical difference when compared to 0 µg/ml, the symbol $^{\#}$ indicates comparison with 100 µg/ml, the symbol $^{\$}$ is used for specific comparisons as indicated in the graph.

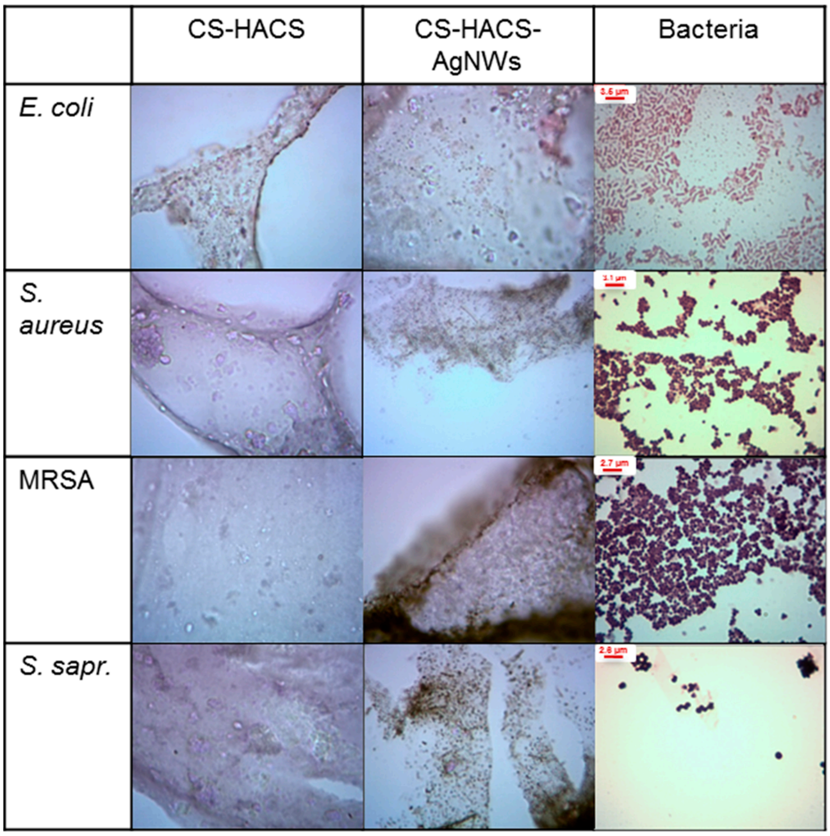

Figure S9. Microscopy images of biofilm formation on CS-HACS, CS-HACS-AgNWs hydrogels and controls after fuchsin (*E. coli*) or crystal violet (*S. aureus*, *MRSA* and *S. saprophyticus*) staining. Magnification 100x.
